# Social support and mental health among health care workers during Coronavirus Disease 2019 outbreak: A moderated mediation model

**DOI:** 10.1371/journal.pone.0233831

**Published:** 2020-05-29

**Authors:** Tianya Hou, Taiquan Zhang, Wenpeng Cai, Xiangrui Song, Aibin Chen, Guanghui Deng, Chunyan Ni

**Affiliations:** 1 Department of Psychology, Naval Medical University (Second Military Medical University), Shanghai, China; 2 Nursing Department, The Affiliated Suzhou Science & Technology Town Hospital of Nanjing Medical University, Suzhou, China; University of Birmingham, UNITED KINGDOM

## Abstract

**Purposes:**

During the outbreak of Coronavirus Disease 2019 (COVID-19) all over the world, the mental health conditions of health care workers are of great importance to ensure the efficiency of rescue operations. The current study examined the effect of social support on mental health of health care workers and its underlying mechanisms regarding the mediating role of resilience and moderating role of age during the epidemic.

**Methods:**

Social Support Rating Scale (SSRS), Connor-Davidson Resilience scale (CD-RISC) and Symptom Checklist 90 (SCL-90) were administrated among 1472 health care workers from Jiangsu Province, China during the peak period of COVID-19 outbreak. Structural equation modeling (SEM) was used to examine the mediation effect of resilience on the relation between social support and mental health, whereas moderated mediation analysis was performed by Hayes PROCESS macro.

**Results:**

The findings showed that resilience could partially mediate the effect of social support on mental health among health care workers. Age group moderated the indirect relationship between social support and mental health via resilience. Specifically, compared with younger health care workers, the association between resilience and mental health would be attenuated in the middle-aged workers.

**Conclusions:**

The results add knowledge to previous literature by uncovering the underlying mechanisms between social support and mental health. The present study has profound implications for mental health services for health care workers during the peak period of COVID-19.

## Introduction

The Coronavirus Disease 2019 (COVID-19) as an unprecedented threat has infected more than 2,800,000 people by 27 April, 2020 [1], which has attracted international attention as a Public Health Emergency of international concern [2]. Considerable countries are confronting escalating pandemics and tremendous burden. The availability of skilled health care workers is the decisive factor in overcoming a viral epidemic [3]. In the most affected areas such as Hubei Province, China where the confirmed cases were first reported, nearly all health care workers work directly with the infectious patients, whereas in the less affected areas, some health care workers directly fight against COVID-19 and others are prepared to fight. However, the bravery of health care workers cannot protect them away from mental health problems during COVID-19. According to the research during the 2003 severe acute respiratory syndrome (SARS) outbreak, health care workers have shown mental health problems with 10% hospital workers reporting higher levels of stress [4,5]. Another study during Ebola outbreak presented that health care workers of Sierra Leone had significant psychological symptoms including depression, interpersonal sensitivity and even paranoid ideation [6]. More importantly, evidence from previous literature showed that the stress levels for high- and low-risk health care workers were equivalent during the outbreak of epidemic [3]. Psychological intervention and mental health services were in need to prevent health care workers from being traumatized as they were emotionally affected during the epidemic [6,7].

Mental health is fundamental to an individual’s overall well-being and absolutely essential to a productive and efficient life. In workplace, mental health problems are found to be associated with plenty of negative influences, such as reduction of efficiency, loss of productivity, disability and absenteeism [8,9]. Given the adverse impacts, it is of great importance to investigate the potential factors and mechanisms that could enlighten the improvement of the mental health and maintenance of productivity of health care workers in the mist of the epidemic. Among all the influential factors, social support has been recognized as one of the protective factors for mental health [10,11]. Thus, the aim of the present study was to replicate the relationship between social support and mental health based on the health care workers during COVID-19 outbreak, and further extend the previous studies by exploring the potential mechanisms in the relationship.

### Social support and mental health

Social support is individuals’ perception or experience in terms of being involved in a social group where people mutually support each other [12]. Previous research has repeatedly emphasized the role of social support in the promotion of mental health [13]. Not only cross-sectional studies [14,15], but also a large body of longitudinal studies emerging recently [13,16] have confirmed the positive association between social support and mental health outcome robustly. Although a substantial number of research found this strong relation can hold across a broad range of samples, such as cancer patients, patients with Multiple Sclerosis, nurse students and so on [17,13,11], a handful of studies have presented different results. A meta-analysis conducted by Ge, Zhao, Liu, Zhou, Guo & Zhang [18] has concluded that for the aged people, mental health was weakly or extremely weakly associated with social support. Fiori et al. [10] has claimed that the emotional support was only related to mental health in females only, not in males.

Therefore, it is vital to note the previous studies have found the relation between social support and mental health based on certain samples, however, whether the conclusion could be duplicated to health care workers during the outbreak of COVID-2019 still remains unexplored. Moreover, the mediating mechanisms (i.e., how social support correlates with mental health?) and moderating mechanisms (i.e., when this relationship is most potent?) underlying the association between social support and mental health also stay largely unknown. Answering these questions could be of vital importance to further understand the mental health of medical workers and advance the more effective interventions to ensure the productivity of health care worker during COVID-19.

Thus, the present research employed a sample of Chinese health care workers during COVID-19 outbreak to explore a conceptual model in which, on the one hand, resilience mediated the association between social support and mental health; On the other hand, the indirect relationships between social support and mental health via resilience were moderated by age group.

### The mediating role of resilience

#### Social support and resilience

Resilience is an individual’s capacity to deal with significant adversity and quick recover [19]. A great many of studies based on various methodologies and samples have provided robust evidence with respect to the association between social support and resilience. Numerous cross-sectional studies have revealed a positive association between social support and resilience [20–22]. In addition, a longitudinal study conducted by Liu, He, Jiang, & Zhou [23] utilized a sample of adolescents from 5.12 earthquake-hit region and confirmed social support as the protective factor of resilience after a one-year followup. Furthermore, a meta-analysis including 14 studies also has concluded that social support, particularly the utilization of the support, could enhance children’s resilience [24]. Thus, it is possible that social supports could enhance the resilience of health care workers.

#### Resilience and mental health

Mental health is the critical component of personal development and growth, which is more than the absence of mental illness [25]. Plenty of empirical studies reach the consensus that resilience exerts an positive effect on mental health [25–27]and the association is presented to be consistent across different samples with diverse background [28–30]. Also, psychological resilience can help protect individuals against mental illness and thrive from the adversity [31].

In fact, a few researchers have investigated the relation between social support, resilience and wellbeing [14,13,15,32]. It is worth noting although the previous studies explored the relation, some treated resilience as a covariate or moderator, while others did analyze resilience as a mediator. However, as far as we know, no research has examined whether the relationship between social support and mental health via social support could be applied to the health care workers who are prepared and fight during the epidemic.

### Age group as a moderator

Although social support may have an impact on mental health indirectly via resilience, not all people with lower resilience suffer from lower level of mental health. Therefore, it is essential to explore the influential factors that could strengthen or attenuate the link between social support, resilience and mental health. This research examined a hypothesis that the resilience-mental health link in the indirect association between social support and mental health would be moderated by age group.

Several studies have concluded the possibility of the receipt of depression treatment diminished as getting older [33,34]. Robb, Haley, Becker, Polivka & Chwa [35] have compared the similarity and difference between younger and older adults in the attitudes towards mental health service, and found younger adults showed more willing to seek mental health services compared with the elder. In addition, Dinapoli, Cully, Wayde, Sansgiry, Yu, & Kunik [36] have examined the role of age in the prediction of mental health service use. The results have presented that younger adults with depression or anxiety disorders were more likely to utilize mental health service compared with the middle-aged adults. The mental health services include mediation, yoga and stress management, which are mainly focusing on the enhancement of resilience [37,38]. In sum, younger adults might be more likely to receive resilience training to improve the mental health than middle-aged adults. Thus, the mental health of younger adults might rely more on resilience than other factors. Furthermore, health care workers during the pandemic are in highly psychological stressing condition when fighting against COVID-2019 outbreak [39]. The middle-aged workers usually have longer length of employment and more working experience than the younger workers. Meanwhile, the odds of participation in the fight against other epidemics before might be higher for the middle-aged in comparison to the younger. All these past experiences would make them less stressful, less anxious, less fearful and better mental health state when facing the epidemic [40]. Taken all together, the mental health of the middle-aged workers would be less dependent on resilience, which indicates the link between resilience and mental health would be attenuated in middle-aged adults than younger adults.

In fact, a meta-analysis including 60 studies has specifically investigated the moderating role of age in the relation between trait resilience and mental health [25]. However, the study just concluded the relation was stronger for adults than children and adolescence, without the comparison between younger adults and middle-aged adults. Unlike the previous research, the current study examined the potential difference between the younger and middle-aged medical workers in the link between resilience and mental health.

### The present study

**Fig 1:**
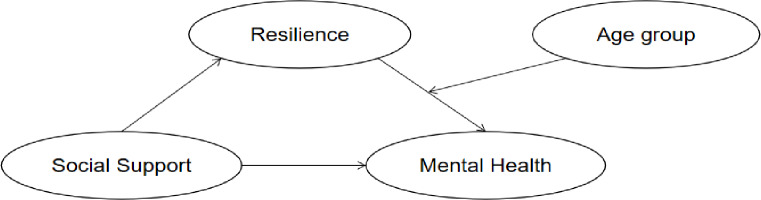
Conceptual model.

Taken all together, the aims of this research were twofold: (a) to examine whether the mediating role of resilience in social support and mental health could be duplicated to the health care workers from a less affected area during the COVID-19 epidemic, and (b) to test whether the relationship between social support and mental health via resilience is moderated by age group. The current study constructed a conceptual model to address both mediation and moderation effects (see Fig 1). Based on the literature review, the following hypotheses were proposed:

Hypothesis 1: Resilience would mediated the relationship between social support and mental health of health care workers during COVID-19 pandemic.
Hypothesis 2: Age group would moderate the indirect association between social support and mental health via resilience such that the resilience-mental health pathway would be stronger in younger age group in comparison with the middle-age group. Given that we suppose age would only moderate the second stage of the mediation path, the present study would call it “a second stage moderation model”.

## Methods

### Participants and procedures

The cross-sectional study was conducted from 1^st^ to 7^th^ February, 2020, which was the peak period of COVID-2019 outbreak in China. The participants were health care workers from local hospitals, community health service centers and government department in Jiangsu Province who participated in the fight against COVID-19. The questionnaires were distributed through Internet. All subjects were given informed written consent before completing the online survey concerning demographic information, social support, resilience and mental health. All the subjects were free to withdraw from the research at any time. The research was approved by the ethics committees of the Second Military Medical University.

A total of 1521 health care workers completed the survey in the present study. Considering the present study was to compare the indirect effect of social support on mental health via resilience between the young and middle-aged heath care workers, participants aged 50 or over were excluded. Finally, 1472 subjects were included in the analysis.

### Measures

#### Social support

The Social Support Rating Scale (SSRS) developed by Xiao was utilized to measure social support [41]. The 10-item scale consists of 3 dimensions including objective support, subjective support and availability. A representative item was “How many close friends do you have to get support and help?”. Higher scores indicate higher levels of social support. The scale has presented impressive validity and reliability in Chinese population [42]. The Cronbach’s alpha for the present study was 0.949.

#### Resilience

The Connor-Davidson Resilience scale (CD-RISC) was used to assess resilience [43]. The 25-item Likert scale consists of five dimensions: (a) personal competence, high standards, and tenacity; (b) trust in one’s instincts, tolerance of negative affect, and strengthening effects of stress; (c) positive acceptance of change and secure relationships; (d) control; (e) spiritual influence [44,45]. Participants rated each item from 0 (not true at all) to 4 (true all the time). The range of total scores is from 0 to 100, with higher scores representing higher levels of resilience. The scale has presented good psychometric properties [43]. In this study, the Cronbach’s alpha was 0.955.

#### Mental health

Symptom checklist 90 (SCL-90) developed by Derogatis and Cleary [46] was administrated to evaluate mental health [47,48]. The 90-item scale is widely applied to measure clinical psychiatric symptoms and differentiate individuals with mental illness from healthy people [49,50]. Each item is rated from 1 (no symptom) to 5 (severe symptom). The higher total scores the participants got, the worse mental health condition they were in. In this research, SCL-90 ≥ 160 was defined as psychological abnormality [51]. The scale has shown good validity and reliability in Chinese population [52]. In this research, the Cronbach’s alpha was 0.983.

### Data analysis

Firstly, the present study calculated the descriptive statistics and bivariate correlations among variables of interest by Statistical Package for Social Science (SPSS) 21.0 for windows. A two-tailed *p*-value smaller than 0.05 indicated the presence of statistical significance. Secondly, structural equation modeling (SEM) conducted by Amos 23.0 through maximum likelihood method was performed to examine the mediating role of resilience in the relation between social support and mental health. The model fit index included root mean square error of approximation (RMSEA), standardized root mean square residual (SRMR), goodness of fit index (GFI) and comparative fit index (CFI). As recommended by previous literature, the values of RMSEA and SRMR smaller than 0.08 and the values of GFI and CFI more than 0.9 indicate an acceptable fit [53]. Bias-corrected bootstrap method was used to examine the significance of mediation effect. Specifically, we used 2000 bootstrap samples and determined the bias-corrected 95% confidence interval. If the confidence does not contain zero, it means the significance of the effects [54]. Finally, the moderated mediation model was tested by using Hayes [55] PROCESS macro (Model 14). The 95% bias-corrected confidence interval from 5000 resamples was generated by bias-corrected bootstrapping method to examine the significance of moderated mediation effect.

## Results

### Preliminary analysis

The sociodemographic characteristics of the participants and the distribution of SCL-90 scores were presented in Table 1. Most health care workers were females (76.5%), middle-aged (55.0%), married (73.0), and reported 12–16 years of schooling (64.9) and less than 10 years of working (61.6). The prevalence of psychological abnormality was 7% among health care workers. The mean ± SD total score of SCL-90 was 110.28 ± 28.89. There were no significant differences in SCL-90 scores associated with gender, age, marital status, years of schooling and years of working. Social support was positively correlated with resilience and age group, and negatively correlated with SCL-90 scores (all *P* < 0.001). Resilience was positively associated with age groups and negatively associated with SCL-90 scores (all *P* < 0.001).

**Table 1:** Sociodemographic characteristics and the distribution of SCL-90 scores.

Variables	Respondents		SCL-90 Scores		*F*/*t*	*P*-value
	n	%	M	SD		
Gender					0.674	0.412
Male	346	23.5	109.12	32.094		
Female	1126	76.5	110.63	29.185		
Age group					1.422	0.233
Younger group (18< 30)	662	45.0	111.31	33.794		
Middle-aged group (30~49)	810	55.0	109.44	26.264		
Marital status					0.682	0.506
Married	1075	73.0	109.76	28.927		
Unmarried	380	25.8	111.81	32.768		
Others	17	1.2	108.94	21.379		
Years of schooling					1.008	0.365
Less than 12 years	294	20.0	110.06	32.593		
12 to 16 years	955	64.9	109.74	27.309		
More than 16 years	223	15.1	112.88	36.12		
Years of working					0.365	0.546
Less than 10 years	900	61.1	110.65	31.894		
10 years or above	572	38.9	109.69	26.446		

### Testing for mediation effect

**Fig 2:**
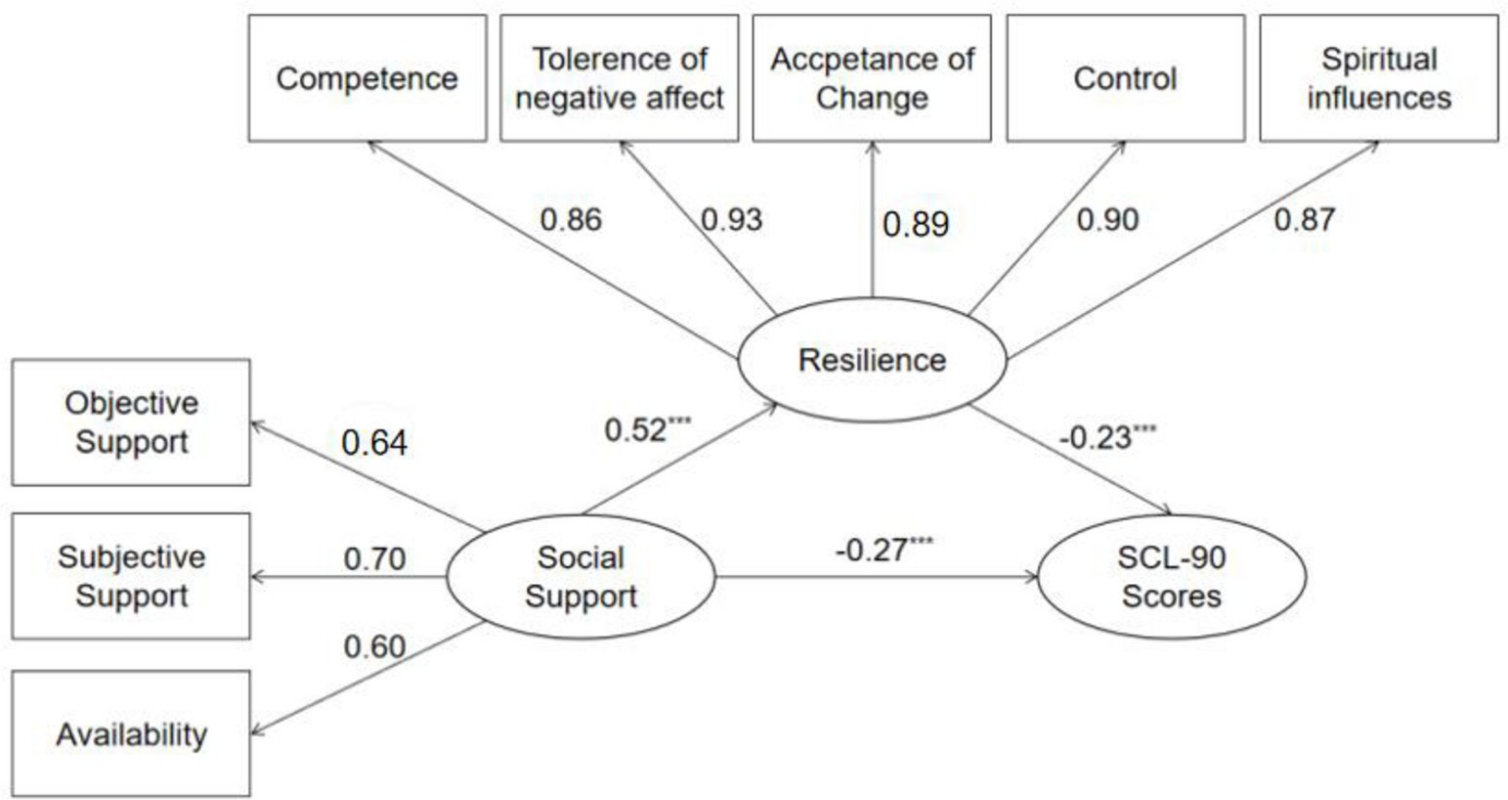
The mediating role of resilience in the association between social support and SCL-90 scores.

Structural equation model was employed to examine the mediating role of resilience. Firstly, the direct path coefficient from social support to SCL-90 scores in the absence of resilience was significant, γ = -0.39, *P* < 0.001. Secondly, the structural equation model regarding the mediating role of resilience in the relationship between social support and SCL-90 scores presented a good fit to the data: χ^2^(25, *N* = 1472) = 115.814, *P* < 0.001; RSMEA = 0.050; SRMR = 0.020; GFI = 0.983; and CFI = 0.989. Thus, resilience partially mediated the relationship between social support and SCL-90 scores (see Fig 2).

A bootstrap procedure conducted to examine the mediation effects. 2000 bootstrapping samples was generated from the original dataset (*N* = 1472) via random sampling. The indirect effect of social support on SCL-90 scores through resilience was -0.120 (SE = 0.019, 95%CI = [-0.156, -0.084], *P* = 0.001). The 95% biased-corrected confidence interval did not contain zero, which verified the indirect relationship between social support and SCL-90 scores via resilience.

### Testing for moderated mediation

**Fig 3:**
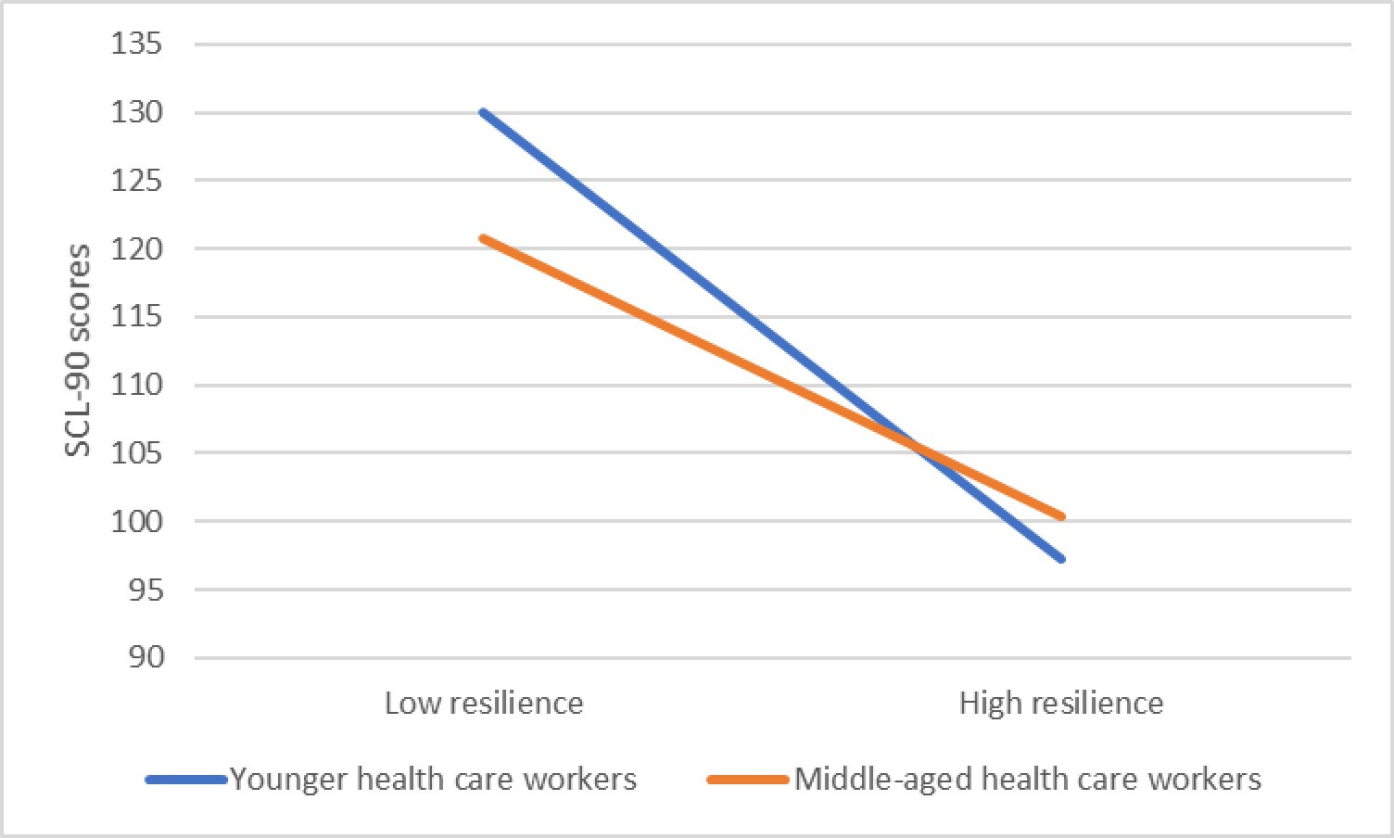
The interaction between resilience and age group on SCL-90 scores.

It has been expected that age group would moderate the second stage of the mediation process. As shown in Table 2, in model 1, social support positively predicted resilience, *β* = 0.408, *P* < 0.001. Model 2 revealed that the effects of resilience on SCL-90 scores was moderated by age group, *β* = 0.126, *P* < 0.01. For descriptive purpose, the present study plotted the relationship between resilience and SCL-90 scores, separately for younger and middle-aged groups (see Fig 3). Simple slope test presented that for subjects from younger group, resilience was significantly and negatively associated with SCL-90 scores, *β*_simple_ = -0.343, *P* < 0.001. For subjects from middle-aged group, resilience was still negatively correlated with SCL-90 scores, but much weaker, *β*_simple_ = -0.217, *P* < 0.001.

**Table 2:** Test the moderated mediation effect of social support on SCL-90 scores.

	Model 1(resilience)		Model 2(SCL-90 scores)	
	*β*	*t*	*β*	*t*
Social support	0.408***	17.168	-0.212***	-7.940
Resilience			-0.469***	-5.944
Age group			0.042	0.853
Resilience * Age group			0.126**	2.614
R2	0.167***		0.169***	
*F*	294.724		74.296	

** *P* < 0.01.

*** *P* < 0.001

The biased-corrected 95% confidence interval for index of moderated mediation was from 0.0065 to 0.0981, which did not contain zero. This further presented that the indirect effects of social support on SCL-90 scores via resilience significantly differed between groups.

## Discussion

Evidence from previous literature has already found health care workers with higher levels of social support are more likely to show higher levels of mental health [56,57]. Nevertheless, issues regarding the underlying mediating and moderating mechanisms and whether this could be applied to health care workers who are fighting with the outbreak of COVID-2019 stay largely unanswered. To our knowledge, the present study is the first to report the effect of social support on mental health based on health care workers from a less affected area during COVID-19 outbreak.

This research built a moderated mediation model to test whether resilience mediated the association between social support and mental health of health care workers and whether this indirect relationship was moderated by age groups. The results showed that (1) the mediating role of resilience in the association between social support and mental health could be replicated to the health care workers during the epidemic; (2) age moderated the indirect link between social support and mental health (resilience-mental health path), with younger workers showing stronger than middle-aged workers.

The prevalence of psychological abnormality was 7% among health care workers in our study, lower than that (17.3%) of Chinese health care workers during the SARS epidemic [58]. A recent study presented the rate of mental abnormality among nurses in Guangdong province was 11.9% during the non-epidemic phase [51]. The difference might be attributed to the fact that Jiangsu province was less affected during the COVID-19 pandemic. Additionally, different instruments and regional differences might also contribute to the discrepancy. Generally speaking, the prevalence of psychological abnormality cannot be neglected and more attention should be paid to address this issue.

### The mediating role of resilience

In line with our hypothesis, resilience mediates the relationship between social support and mental health of health care workers during the epidemic. This is aligned with previous research based on different samples [13,16]. This study is the first to explore the relation with the focus on the population who are facing the emergency events of the public health.

This finding can be explained by the “buffer” hypothesis developed by Cohen and Wills [59], which revealed the buffering effect of social support on the impact of stress upon mental health. The previous research has highlighted that finding effective approaches to deal with stress is of great importance in positive health outcomes [12]. Social support could protect individual from stressful conditions and poor health state [60]. Meanwhile, individuals with higher levels of social support might be more inclined to believe that they could get the help needed when facing the stressful event regarding the outbreak of the epidemic. This notion would enhance their beliefs to deal with the adversity and difficulty in the battle with COVID-19, which further leads to the higher levels of resilience [61]. In addition, the reports of previous literature have demonstrated that resilience, as one kind of personal resources, also buffered the impact of stress on mental health [62–65]. Taken all together, resilience plays a mediating role in the association between social support and mental health for the health care workers fighting with the epidemic.

### The moderating role of age group

The findings also revealed the moderating role of age groups in the association between resilience and mental health of the health care workers. In the past research, a small handful of previous studies have found that age moderated the link between resilience and mental health by the comparison between younger adults and older adults (usually age 50 and over) [66,67], whereas some other researchers focused on the contrast between the adults and minors [25]. However, these studies neglected the potential differences between the younger adults and middle-aged adults. Unlike the past research, this study took the potential difference between younger and middle-aged health care workers into consideration innovatively.

Consistent with our hypothesis aforementioned, the increase in age (from young adults to middle-aged adults) attenuated the relationship between resilience and mental health. Specifically, the younger health care workers showed stronger association between resilience and mental health compared to the middle-aged ones. This result might be interpreted by Erikson’s theory of life cycle development [68], which postulates eight stages of human life. Younger adults belong to stage VI with the emphasis on intimacy, while middle-aged adults are attributed to stage VII focusing on generativity. Erikson’s generativity stage is similar to self-actualization in Maslow's hierarchy of needs [69,70]. Hence, compared with younger adults, the mental health of middle-aged adults relies less on resilience but other factors, such as the feeling of self-fulfillment, personal growth and so on.

### Implications

The findings of this research have profound implications since the data were collected during the peak period of COVID-19 in China, which is the stage other countries are currently experiencing. First, the results stress the crucial role of social support in mental health. During the outbreak, the maintenance of mental health of health care workers is crucial for the productivity and work efficiency. Therefore, it is of great importance to provide a full range of social support including instrumental support, emotional support and so on. Second, the findings also highlight the potential value of resilience-focused intervention in health care workers. The resilience-focused mental health promotion program usually contains coping skills, stress management, positive attitude and so on [71]. Finally, the indirect link between social support and mental health via resilience is stronger in younger adults, which implies we should give priority to younger health care workers with respect to resilience-boosting intervention. Meanwhile, we also need to find other influential factors of mental health for middle-aged health care workers.

### Limitations and contributions

Several study limitations must be noted. First, the current study employed a cross-sectional design since health care workers who have consecutively worked for several days would be replaced by others, which made it hard to revisit them after a certain period. However, the cross-sectional design is insufficient to infer the causality in terms of the relationship analyzed. Also, as existing literature showed, social support and resilience might influence each other reciprocally [23,72], the reverse causality cannot be ruled out. Future research could conduct longitudinal study to further explore the moderated mediation model. Second, the study used convenient sampling and all data were collected through self-report, which undermined the generalization of the results. The participants in our study were all from Jiangsu Province, China, which was geographically limited for a wider generalization. Future research could test the relation based on more typical samples from multiple regions and manage to collect data from multiple sources (e.g., colleagues). Finally, mental health is a complex concept, which could be influenced by numerous factors. Social support and resilience could just explain a limited part of mental health. Sleep quality and fatigue might be two important factors of mental health, but we failed to investigate. During the COVID-19 outbreak, health care workers had to work excessive hours and suffered from disrupted circadian rhythms, which would contribute to fatigue and undermine sleep quality. Hence, future study might focus on a more integrated model of mental health with diverse influential factors.

In spite of the limitations, the current study contributes to the previous literature theoretically and practically. Theoretically, this study adds knowledge to the previous research by exploring the moderated mediation model, which would help further understand the relationship between social support and mental health. Practically, the findings are essential for the maintenance of mental health of health care workers during the outbreak of COVID-2019.

## Conclusion

In conclusion, this study presented the protective role of social support in mental health among health care workers. Moreover, resilience could be one of the pathways through which social support contributes to mental health. Furthermore, the effect of social support on mental health via resilience is attenuated in middle-aged health care worker compared with the younger ones.

## Supporting information

S1 File (SAV)

S1 Data STROBE statement—checklist of items that should be included in reports of *cross-sectional studies*. (DOC)
